# Evolutionary Limitation and Opportunities for Developing tRNA Synthetase Inhibitors with 5-Binding-Mode Classification

**DOI:** 10.3390/life5041703

**Published:** 2015-12-08

**Authors:** Pengfei Fang, Min Guo

**Affiliations:** 1 State Key Laboratory of Bioorganic and Natural Products Chemistry, Shanghai Institute of Organic Chemistry, Chinese Academy of Sciences, 345 Lingling Road, Shanghai 200032, China; fangpengfei@sioc.ac.cn; 2 Department of Cancer Biology, The Scripps Research Institute, Scripps Florida, 130 Scripps Way, Jupiter, FL 33458, USA

**Keywords:** aminoacyl-tRNA synthetase (aaRS), inhibitor, evolution, protein-ligand interaction, structure conservation, species specificity

## Abstract

Aminoacyl-tRNA synthetases (aaRSs) are enzymes that catalyze the transfer of amino acids to their cognate tRNAs as building blocks for translation. Each of the aaRS families plays a pivotal role in protein biosynthesis and is indispensable for cell growth and survival. In addition, aaRSs in higher species have evolved important non-translational functions. These translational and non-translational functions of aaRS are attractive for developing antibacterial, antifungal, and antiparasitic agents and for treating other human diseases. The interplay between amino acids, tRNA, ATP, EF-Tu and non-canonical binding partners, had shaped each family with distinct pattern of key sites for regulation, with characters varying among species across the path of evolution. These sporadic variations in the aaRSs offer great opportunity to target these essential enzymes for therapy. Up to this day, growing numbers of aaRS inhibitors have been discovered and developed. Here, we summarize the latest developments and structural studies of aaRS inhibitors, and classify them with distinct binding modes into five categories.

## 1. Introduction

Aminoacyl-tRNA synthetases (aaRSs) are essential enzymes for all cellular life, playing a central role in the translation of the genetic code [1,2]. Despite the differences in the substrate amino acids, all aaRSs catalyze the shared two-step process to attach specific amino acids to their corresponding tRNAs [1]. In the first step, the enzyme binds a specific amino acid and an ATP molecule, and catalyzes the covalent linkage between the 5′-phosphate group of ATP and the carboxyl end of the amino acid to form the aminoacyl-adenylate (aa-AMP) intermediate. After this step, amino acid is activated by the hydrolysis of ATP, and the energy is trapped in the aa-AMP molecule, which remains associated with the enzyme. In the second step, aaRS binds its cognate tRNA, and further transfer amino acid from the aa-AMP to either 2′-OH or 3′-OH of the evolutionarily invariant 3′-adenosine terminus of the tRNA molecule. The energy in the aa-AMP is thereby used to make a new covalent bond, which results in the formation of aa-tRNAs.

Because they define the linkage between the amino acid and tRNA, aaRSs are critical for the fidelity of genetic code. Their mistakes will lead to undesired mutations in protein sequences during translation, which may result in mis-folding and/or inactivation of proteins. While these mistakes can happen from two sides, the selection of tRNA or the selection of amino acid, tRNA usually is less of a concern as tRNA offers more choices of interactions for aaRS. During the long co-evolution, tRNAs have developed anticodon arm, acceptor arm, and variable loop for the discrimination by aaRSs, and aaRSs have evolved anticodon-binding domain, 3′-tRNA binding site, and other tRNA binding extensions for the recognition of specific regions of tRNAs [3,4,5,6,7,8,9]. Extra putative ‘anticodons’ on the sidearm loops of mitochondrial tRNAs may also be recognized by aaRSs and affect translation activity [10,11,12]. For most amino acids, while the error rate can be maintained below 10^−4^–10^−5^ [13,14,15], however, when the difference of the side chains is too small or the *in vivo* concentrations of similar amino acids are too different, the error rate will increase [16,17]. To conquer this problem, those aaRSs with amino acid selection problem evolved editing domains, where the charged tRNA goes through a proofreading process before being delivered to protein synthesis [18]. Editing domain ensures the accuracy by exclusion of correctly charged aa-tRNA and by binding and hydrolyzing the mischarged aa-tRNA. Cost minimization hypothesized that the evolution of editing domain of aaRS ensures an “expensive” amino acid to used only where it is unavoidable [19]. While compensation of tRNA misacylation by codon mismatch on ribosome is possible [20], the initiative editing function of aaRS plays an essential role in the fidelity of translation.

aaRSs exist in all living cells and protein-making organelles, such as mitochondria, chloroplast in plant, and apicoplast in parasitic apicomplexa. Although all aaRSs catalyze the aminoacylation reaction, they mostly work independently with every one of them being essential. Therefore, aaRSs provide ~20 distinct targets in bacteria (some don’t have GlnRS), and approximately doubled amount of targets in eukaryotic pathogens, for example: 37 in *Plasmodium falciparum* (*P. falciparum*), including cytoplasmic, mitochondrial, and apicoplastic aaRSs [21]. The high evolutionary divergence across species makes it possible to target the specific pathogenic microorganisms [22,23].

## 2. aaRSs as Target for Disease Therapy

Not only the pathogen aaRSs can be targeted to cure infections, the human aaRSs can also be targeted for disease therapy. In human cells, there are 19 cytoplasmic aaRS polypeptides with 20 aaRS activities (The GluRS and ProRS are naturally fused into a bifunctional GluProRS encoded by one gene). Human mitochondria have 17 mitochondria specific aaRSs, shares two cytoplasmic aaRS (GlyRS, and LysRS) [24,25], but lacks GlnRS, whose activity is compensated by GluRS and Glu-tRNA^Gln^ amidotransferase in mitochondria [26]. Mutations or miss-regulation of these aaRSs are associated to several human diseases [27,28,29]. Mutations Lys81Thr and Arg751Gly in AlaRS reduce aminoacylation efficiency and are found in early infantile epileptic encephalopathy patients [30]. Eight mutations in AspRS were reported to cause hypomyelination with brain stem and spinal cord involvement and leg spasticity [31]. HisRS mutation Tyr454Ser is involved in Usher syndrome type 3B [32]. LysRS mutations Tyr145His and Asp349Asn are potential causes of autosomal recessive deafness [33]. Two LeuRS mutations outside of the active center Lys82Arg and Tyr373Cys are associated to infantile hepatopathies [28,34]. MetRS mutations Phe370Leu and Ile523Thr are found in infantile liver failure syndrome 2 patients [35]. GlnRS mutations are found to be involved in progressive microcephaly [36]. ArgRS mutations Asp2Gly and Arg512Gln are involved in hypomyelinating leukodystrophy [37]. Over 27 mutations of cytoplasmic GlyRS (15), TyrRS (3), LysRS (3), MetRS (2) and AlaRS (4) are associated to the Charcot-Marie-Tooth diseases [27,38,39,40]. Most of these mutations locate at the catalytic domains of these aaRSs, though only 20% of these mutant proteins affect aminoacylation activity [28]. Potential gain-of-function of the mutants or a loss-of-function of unknown secondary activities of these aaRSs have been speculated [27,41,42,43]. The mechanism for aaRS mutants to cause the disease remains unknown and these diseases are currently not curable. More mutations in mitochondrial aaRSs, than cytoplasmic ones, have been associated to human diseases [27,28], such as, mtAlaRS mutations in progressive leukoencephalopathy and combined oxidative phosphorylation deficiency [44,45], mtAspRS mutations in leukoencephalopathy [46], *etc*. Some of these mitochondrial aaRSs disease-causing mutations decreased the aminoacylation activity [45,46,47,48,49,50,51,52,53]. Developing drug molecules to rescue the aminoacylation activity, the amino acid transportation to mitochondria, or the stability of the mutant aaRSs are possible strategies to treat these diseases.

The involvement of aaRSs in human cancer is also reported [54]. Increased PheRS activity has been reported in myeloid leukemia [55]. Activity of MetRS from homogenates of tumors was increased by four fold in human colon cancer comparing to adjacent normal tissue [56], GlyRS up-regulation was reported in papillary thyroid carcinoma [57], and LysRS is overexpressed in breast cancer [58]. Amino acids play essential roles in cells, as they are intermediate metabolites for other biosynthetic reactions and the major source for protein synthesis [59]. Their cellular concentrations are sensed by two important pathways, mTOR and GCN2 pathways, which are both involved in tumorigenesis [60,61]. Amino acid deprivation, resulting in inhibition of protein synthesis and apoptotic cell death in tumor cells, has been proposed as a novel approach for cancer therapy [62,63]. Metabolic enzymes, which deplete amino acids in blood, have been used or tested for certain kinds of cancer treatment. For example, asparaginase is marketed as a drug for the treatment of acute lymphoblastic leukemia and is also used in some mast cell tumor protocols [64]. Arginase and arginine deiminase are being actively researched for melanoma, prostate cancer, renal cell carcinoma, *etc.* [65]. The therapeutic benefit of amino acid deprivation therapy can be affected by three aspects: amino acid source in diet, efficiency of amino acid clearing enzyme, and compensatory protein turnover. Beside, this therapy can be limited to certain types of amino acid for certain types of cancer, where the corresponding amino acid is semi-essential or conditionally essential. While the roles of aaRSs in cancer remain largely unclear, suppression of aaRSs in general may serve as an alternative way of amino acid depletion therapy for cancer treatment.

aaRSs are also involved in autoimmune diseases in two aspects. To date, eight different human cytoplasmic aaRSs (IleRS, HisRS, GlyRS, AsnRS, AlaRS, ThrRS, TyrRS, and PheRS) have been identified as autoantigens in human anti-synthetase syndromes [66]. The molecular pathway that initiates and propagates this autoimmune response and the specific role of the antisynthetase antibodies in the pathogenesis of this syndrome are presently unknown. The human ProRS (as part of the dual GluProRS) inhibitor Halofuginone (HF) received FDA’s orphan drug designation for the treatment of scleroderma, which is a chronic systemic autoimmune disease affecting skin and internal organs. HF triggers the amino acid response (AAR) pathway, selectively blocks IL-23-mediated Stat3 signaling, and thereby inhibits the development and progression of Th17 cell, which plays an important role in autoimmune disease [67]. Particularly HF does not affect other kinds of T cells in normal immune function [68]. These studies raise possibility that other inhibitors targeting aaRSs may be developed into therapies for the treatment of autoimmune diseases.

In addition to their multiplexed roles for translation, aaRSs regulates many other cellular pathways [38]. For example, TrpRS can be induced and secreted under IFN-γ stimulation. By removal of the appended N-terminal domain, TrpRS is activated and then binds to vascular endothelial cadherin on the surface of endothelial cells and inhibits the formation of endothelial cell–cell junctions that are critical for vasculature development [69]. After stimulation with IFN-γ, GluProRS is released from the Multi-aminoacyl-tRNA Synthetase Complex (MSC), where it becomes part of the GAIT complex (γ-interferon-activated inhibitor of translation complex) and silences translation by binding to a stem-loop structure (GAIT element) in the 3’-untranslated region of one or more specific mRNAs that function in pathways for inflammation and iron homeostasis [70]. In mast cell, antigen activation triggers the MAPK-dependent phosphorylation of LysRS, which promotes the nucleus translocation of LysRS, enhances its activity for Ap_4_A synthesis, thereby boost the transcription of microphthalmia-associated transcription factor (MITF) target genes [71]. In breast cancer cells, LysRS forms a membrane-anchored complex with the laminin receptor 67LR and promotes the cell migration and metastasis of the tumor [72]. LysRS could also be secreted as a cytokine that binds to macrophages and peripheral blood mononuclear cells to activate their migration and TNF-α production [58].

**Figure 1:**
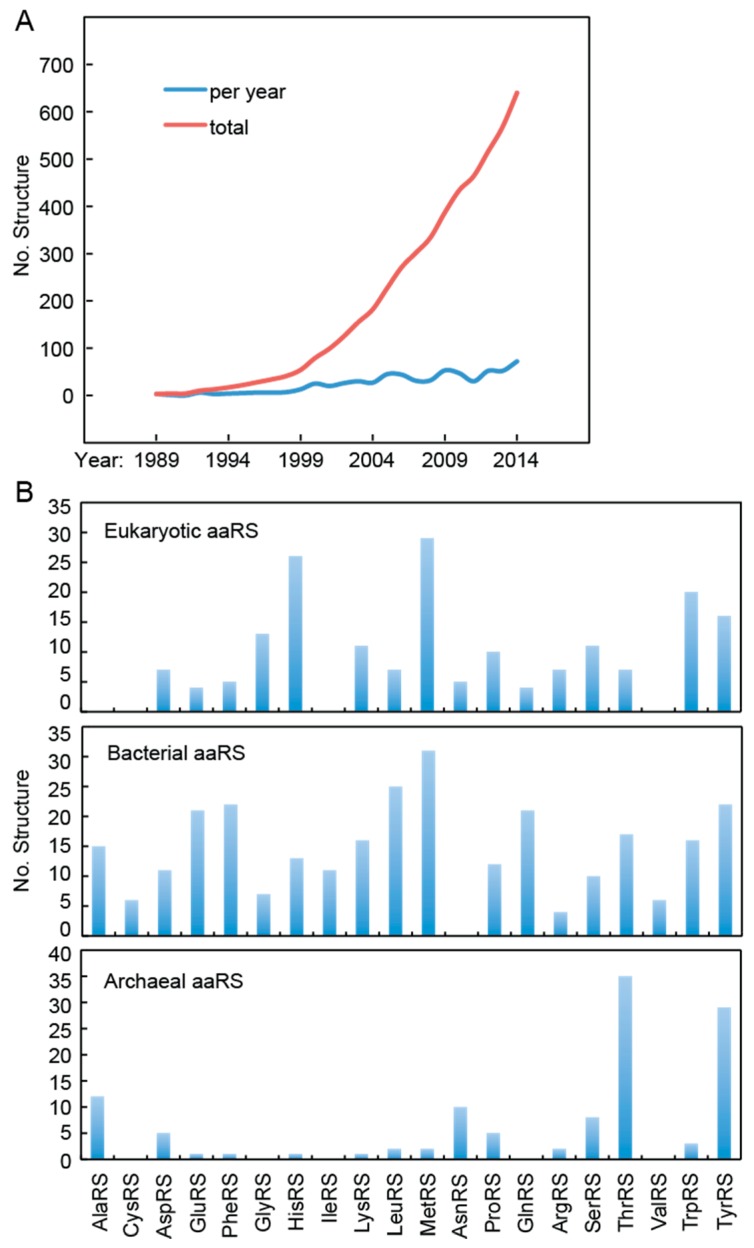
Statistics of aaRS structures from protein data bank. (**A**) Number of deposited aaRS structures was plotted by addition per year (blue) or by total number (red). (**B**) Statistics of aaRS structures from each domain of life.

During the long history of research, plenty of aaRS structures from all three domains of life are solved. Since the first structure published in 1976 [73], the number of the released AARS structures in PDB is still exponentially growing (Figure 1A). Each of the 20 common aaRS families at least has prokaryotic homolog structures available. For the eukaryotic kingdom, structures for 16 aaRS families have become available, except four aaRS families including AlaRS, CysRS, IleRS, and ValRS (Figure 1B). The rich structural information has built up a great platform for structure-based drug design and computational simulation based drug screening against aaRSs.

## 3. Classification of aaRS Inhibitors

Because of their vital role in organisms, and the long history of research, inhibitors targeting aaRSs translational or non-translational functions are being actively searched for drug development [68,72,74,75,76,77]. Up to date, 3 aaRS inhibitor medicines have been approved for human or veterinary use. They are IleRS inhibitor mupirocin, ProRS inhibitor HF, and LeuRS inhibitor AN2690. Mupirocin is effective against Gram-positive bacteria, used as a topical treatment for bacterial skin infections, and is one of the World Health Organization listed essential medicines [78]. HF is a synthetic halogenated derivative of febrifugine, an active ingredient of Chinese medical herb *Dichroa febrifuga*. It was approved for veterinary use against coccidiosis in European Union in 1999 [79], and received orphan drug designation for the treatment of human scleroderma from FDA in 2000 [80]. The boron-based compound AN2690 is a tropical antifungal medication for the treatment of onychomycosis and was approved in 2014 [81]. These medicines clinically validated aaRSs as valuable targets for drug development. A complete list of characterized aaRS inhibitors are summarized in Table 1. A general analysis of aaRS inhibition, particularly how the nature of aaRS allows specific recognition of these individual inhibitors, has not been carefully performed. Here we analyzed these known aaRS inhibitors with their aaRS families, and categorize them into five general groups based on their different binding mechanisms.

**Table 1:** Classification of aaRS inhibitors.

Chemicals	Acylation				Editing	Other	Notes
	ATP	aa	tRNA	auxiliary			
**Class I. Single Active Site Inhibitor**							
AMPCPP and AMPPNP	√						AMPCPP and AMPPNP are non/slow hydrolyzable ATP analogues.
Cladosporin (CP)	√						CP partially mimics ATP and specifically inhibits *Plasmodium falciparum* LysRS [82].
Chem1781	√						Chem1781 is one of the 15 chemical fragments, which bind *Trypanosoma cruzi* HisRS [83].
Amino alcohols (aa-ol) and Amino acid hydroxamates (aa-Hdx)		√					aa-ols and aa-Hdxs are non-reactive amino acid analogues.
Non proteinogenic amino acids		√					Some non-proteinogenic amino acids can compete with proteinogenic amino acid for the binding and reaction.
Resveratrol		√					Resveratrol is a widely used nutrition supplement, inhibits human TyrRS aminoacylation, but activates TyrRS’ non-translational function that stimulates PARPI [84].
2-Aminoquinolin-8-ol				√			This is a potential allosteric aaRS inhibitor, which traps *Clostridium difficile* MetRS in a “non-aminoacylation conformation” [85].
AN2690					√		AN2690 is a broad-spectrum antifungal compound recently approved for onychomycosis treatment. It adducts tRNA at the editing site of fungal LeuRS, inhibits aminoacylation activity by disrupting tRNA turn over [74].
Puromycin					√		Puromycin is a well-known ribosome-targeting antibiotic. It can also bind to PheRS editing site [86].
**Class II. Dual Active Site Inhibitor**							
aa-AMS(s), aa-ol-AMP(s), and aa-Hdx-AMP(s)	√	√					These are reaction intermediate aa-AMP analogues with high binding affinity to aaRSs.
Quinazoline derivatives of Thr-AMS	√	√					These inhibitors were developed from Thr-AMS with improved selectivity for bacterial ThrRS [87].
Thiazole sulfametes	√	√					These compounds contain a thiazole moiety instead of adenine in aa-AMS [88].
Microcin C	√	√					The *in vivo* processed product of Microcin C is a non-hydrolyzable aspartyl-adenylate analogue that inhibits AspRS in bacteria [89].
Agrocin 84	√	√					The *in vivo* processed product of Agrocin 84 is a non-hydrolyzable leucyl-adenylate analogue that inhibits LeuRS in *Agrobacterium tumefaciens* [90].
Mupirocin	√	√					Mupirocin structurally mimics Ile-AMP, inhibits Gram-positive bacteria growth, and is the first approved aaRS inhibitor drug for human [75].
Febrifugine		√	√				Febrifugine is a bioactive natural product extracted from root of the hydrangea *Dichroa febrifuga* Lour used in traditional Chinese medicine, inhibiting ProRS and possessing antimalarial activity [91].
Halofuginone (HF)		√	√				HF is a halogenated derivative of febrifugine, inhibits ProRS in mammalian system, induces antifibrotic activities in fibroblasts through inhibition of T helper 17 cell differentiation [68],[92],[93]. HF has obtained FDA’s orphan drug designation.
Phenyl-thiazolylurea-sulfonamides		√	√				This is a novel class of specific bacterial PheRS inhibitors [94].
REP3123 and its analogues		√		√			This is a novel class of bacterial specific MetRS inhibitors [95].
**Class III. Triple Active Site Inhibitor**							
Triple active site inhibitors	√	√	√				This is a conceptual class of aaRS inhibitors proposed in this paper. This kind of inhibitors may be useful for ArgRS/GluRS/GlnRS, whose 3 substrates bind to aaRSs synergistically.
**Class IV. Multi-Site Inhibitor**							
Borrelidin (BN) and its analogues.	√	√	√	√			BN has a unique 18-member macrolide ring structure, inhibits bacterial and eukaryotic ThrRS through an induced-fit mechanism, and occupies all three substrate-binding sites and an extra area in the active site cavity, inducing a significant conformational change to ThrRS [96]. BN derivative BC220 showed significantly improved druggability as an antimalarial [97].
**Class V. Non-Translational Function Inhibitor**							
YH16899						√	YH16899 inhibits non-translational function of LysRS for cancer cell migration [72].
**Ungrouped Inhibitors**							
4-(2-Nitro-l-propenyl)-1,2-benzenediol							This is a potential AlaRS inhibitor obtained from structural-based virtual screening [98].
Spirocyclic furan and pyrrolidine inhibitors							These inhibitors were obtained from high throughput screening (HTS) to inhibit *Enterococcus faecalis* and *Staphylococcus aureus* PheRS [99].
Pyrazoles							HTS identified a series of pyrazoles, which selectively inhibit bacterial MetRS [100].
Oxazolone-dipeptides							Two HTS identified oxazolone-dipeptides and their analogues showed selective inhibition on bacterial MetRS [101].

In the header row, “ATP”, “aa”, “tRNA”, “auxiliary”, “editing”, and “other” indicate the occupancies of corresponding areas/pockets by aaRS inhibitors. “ATP” refers to ATP binding site; “aa” refers to amino acid binding site; “tRNA” refers to tRNA A76 binding site; “auxiliary” refers to area near by acylation active center but outside of ATP, aa, and tRNA A76 binding pockets; “editing” refers to editing site; and “other” refers to areas differ from the previous four positions.

### 3.1. Single Site Inhibitors

**Figure 2:**
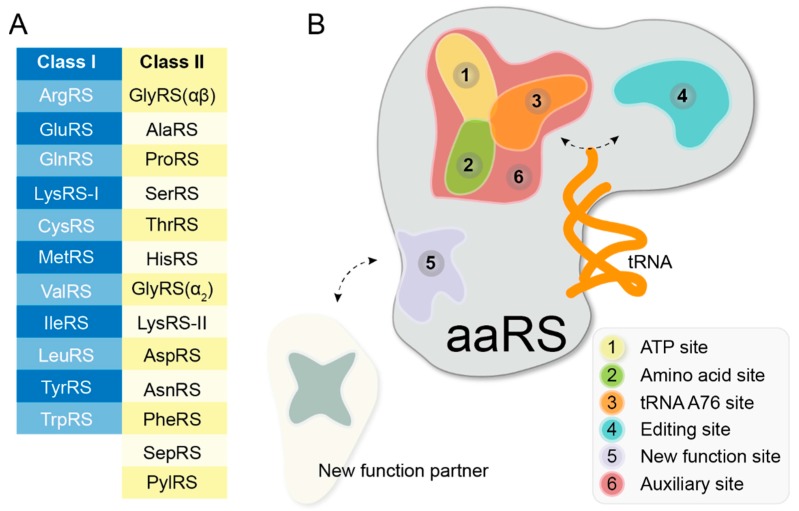
Potential druggable sites on aaRSs. (**A**) List of two classes of the 24 known aaRSs. (**B**) Cartoon of potential druggable sites on aaRSs. ATP site, amino acid site, tRNA A76 site, editing site, extra/auxiliary area nearby the active center, and non-translational function site are colored in yellow, green, orange, green cyan, red and light blue, respectively.

The two-step reaction of aminoacylation involves the binding of amino acid and activation of ATP, followed by a transfer of the activated amino acid to the 3′-end of appropriate tRNA [102]. To carry out aminoacylation reaction, each aaRS has three substrate-binding sites in their active center, plus one additional editing site (for some aaRSs), which proofreads the mischarged tRNA. Besides, extra pockets on aaRS surface can either be constantly present or induced by the binding of other partners. All these different sites can be target for small molecule compounds (Figure 2).

#### 3.1.1. ATP Site Inhibitors

**Figure 3:**
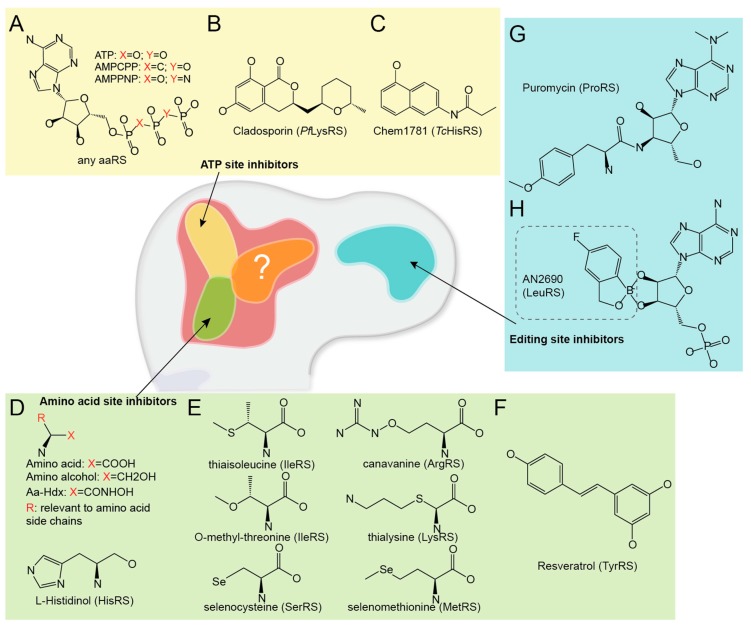
Single site inhibitors of aaRS. (**A**–**C**) ATP site inhibitors of aaRS: (**A**) chemical structures of ATP and ATP analogues; (**B**) chemical structure of Cladosporin (CP); and (**C**) chemical structure of a representative *Trypanosoma cruzi* HisRS ATP site binding fragment Chem1781. (**D**–**F**) Amino acid site inhibitors of aaRS: (**D**) chemical structures of amino acid and amino acid analogues; (**E**) chemical structures of five non-proteinogenic amino acids and selenocysteine; and (**F**) chemical structure of resveratrol. (**G**–**H**) Editing site inhibitors of aaRS: (**G**) chemical structure of PheRS editing site binder puromycin and (**H**) chemical structure of LeuRS editing site inhibitor AN2690.

This group contains mostly ATP mimicking compounds (Figure 3A–C). The non-hydrolysable ATP analogues, such as α,β-Methyleneadenosine 5′-triphosphate (AMPCPP), and β,γ-Imidoadenosine 5′-triphosphate (AMPPNP), bind to the ATP binding site with almost identical binding mode as ATP. They are widely used as replacements of ATP in structural research to visualize the pre-reaction state [103]. Since these chemicals may have similar binding affinity to aaRSs as ATP and would bind other ATP-binding proteins as well, the therapeutic use is limited.

Recently, a potent *P. falciparum* LysRS (*Pf*LysRS) inhibitor cladosporin (CP) was discovered as an ATP-mimetic compound [104]. CP contains two moieties: a (6,8)-dihydroxyl-isocoumarin moiety and a methyltetrahydropyran moiety (Figure 3B). The isocoumarin part mimics the adenine in ATP, while the pyran part replaces the ribose in ATP with hydrophobic interactions to the enzyme instead of polar interactions for ribose. Therefore, unlike AMPCPP or AMPPNP, CP only partially mimics ATP molecule for interaction [82]. Its difference with ATP turns out to be important for the family specificity of CP against LysRS versus other evolutionarily related class II aaRSs [82]. The pyran moiety forms hydrophobic interactions with three small residues (Ser344, Gly554, and Gly556) at the bottom of the ATP binding pocket of *Pf*LysRS, while the corresponding residues in other class II aaRSs are either too large and clash with CP or too small to form the interaction [82]. CP inhibits only eukaryotic LysRS but not bacterial LysRS where the Ser344 is replaced by a larger Met residue [104]. The most interesting feature of this chemical is the high species specificity against *Pf*LysRS with a 1000-fold lower IC_50_ comparing to other eukaryotic LysRS’s including human LysRS [104]. This species specificity is independent of the active site binding residues, but connected to the overall structure stability of the *Pf*LysRS. In the presence of substrate lysine amino acid, CP leads to specific stabilization of *Pf*LysRS, but not human LysRS [82]. Although the poor oral activity preventing CP from practical antimalarial usage, the structure of CP provides a novel scaffold promising for further development [104]. This case also suggests the possibility to generate specific inhibitor of aaRS by targeting the universal conserved ATP binding site.

Most recently, a study using fragment-based crystallographic cocktail screening identified 15 chemical fragments, which can bind to a hotspot in *Trypanosoma cruzi* HisRS (*Tc*HisRS) [83]. One representative fragment Chem1781, *N*-(5-hydroxynaphthalen-2-yl)propanamide, is shown in Figure 3C. The hotspot overlaps with the ATP binding site of *Tc*HisRS [83]. The ATP binding site of *Tc*HisRS exhibits a significant divergence comparing to human HisRS, suggesting that the growing of these fragments into specific *Tc*HisRS targeting drugs may be possible for the treatment of American trypanosomiasis (Chagas disease) [83].

#### 3.1.2. Amino Acid Site Inhibitors

Amino alcohols (aa-ol) and amino acid hydroxamates (aa-Hdx) can bind to the amino acid binding site of aaRSs, and inhibit aminoacylation reaction (Figure 3D). Histidinol (0.1 mM) is able to inhibit protein synthesis in cultured HeLa cells by 50%, when 0.005 mM histidine is present in the culture medium [105]. The mild inhibition of HisRS by histidinol generated similar cellular effect as histidine deprivation [106]. More interestingly, _L_-histidinol protects normal cells from various anticancer drugs, such as 1,3-bis(2-chloroethyl)-1-nitrosourea, cisplatinum, 5-fluorouracil and cytosine arabinoside, while enhancing the toxicity of the same agents in drug-sensitive and multidrug-resistant tumor cells by a mechanism dependent upon protein synthesis inhibition [107].

Some known antibiotic amino acid analogues, such as thiaisoleucine, *O*-methyl-threonine, thialysine, canavanine, *etc.*, can also bind to the amino acid site in aaRSs in the same way as proteinogenic amino acids (Figure 3E). Cell growth inhibition activity of these chemicals is a combined effect of inhibition of aminoacylation of normal amino acids and their mis-incorporation into polypeptides resulting in accumulation of inactive proteins [108]. Besides, two selenium-containing amino acid, selenomethionine and selenocysteine exist naturally. *In vivo*, selenomethionine is randomly incorporated at site of methionine, and is readily oxidized. Incorporation of selenomethionine into proteins into place of methionine has only a limited effect on protein structure, therefore widely aids the structure elucidation of proteins by X-ray crystallography using single- or multi-wavelength anomalous diffraction (SAD or MAD) [109]. Unlike selenomethionine, selenocysteine (Sec) is a real proteinogenic amino acid used. It is charged by SerRS to tRNA^Sec^, existing in all kingdoms of life as a building block of selenoproteins, and is essential for organisms [110].

The red wine extract resveratrol is reported to extend lifespan, provide cardio-neuro-protective, anti-diabetic, and anti-cancer effects, and is widely used as daily supplement (Figure 3F) [111]. The comprehensive health benefit of resveratrol may be attributed to its ability to target multiple cellular pathways and by interacting with multiple targets including quinone reductase 2, transthyretin, leukotriene A4 hydrolase, troponin C, sirtuin 1, sirtuin 3, sirtuin 5, peroxisome proliferator-activated receptor, methionine adenosyltransferase, estrogen receptor, *etc.* [112,113,114,115,116,117,118,119,120]. Recently, it was found to be an effective TyrRS inhibitor with an inhibition constant (Ki) value of 22 µM by inserting its phenolic ring into the tyrosine-binding site on TyrRS [84]. The small molecule nullifies the catalytic activity and redirects TyrRS to a nuclear function, stimulating NAD+-dependent auto-poly-ADP-ribosylation of poly-(ADP-ribose) polymerase 1 (PARP1) [84].

There is no aaRS inhibitor that only binds to tRNA A76 binding site to our knowledge; possibly because the potential tRNA A76 binders may also bind to ATP binding site with better affinity.

#### 3.1.3. Editing Site Inhibitors

The editing site is another hotspot for developing aaRS inhibitors. Several substrate analogs mimic the 3′-end of mischarged tRNA to bind the editing domain of aaRSs [121,122,123,124,125]. The famous aminonucleoside antibiotic puromycin is widely used in biochemistry and cell biology laboratories, for its activity to cause premature chain termination during translation in ribosome [126]. The chemical resembles the 3′ end of aminoacylated Phe-tRNA (Figure 3G), enters the A site and transfers to the growing chain, causing the formation of a puromycylated nascent chain and premature chain release [127]. Interestingly, this resemblance of aminoacylated Phe-tRNA also makes puromycin a PheRS editing site binder with a dissociation constant (Kd) of 300 µM, suggesting a way that the mischarged Tyr-tRNA^Phe^ might bind [86].

AN2690 is a broad-spectrum antifungal compound recently approved for onychomycosis treatment [81]. It is the second approved aaRS inhibitor drug for human (Mupirocin was approved in 1987; HF is currently limited to veterinary use) and the first aaRS editing site-targeting drug. When binds to the fungi LeuRS, AN2690 reacts with the co-bound tRNA^Leu^ thus forming a covalent tRNA^Leu^-AN2690 adduct in the editing site [74]. The adduct formation is mediated through the boron atom of AN2690 attacking the 2′- and 3′-oxygen atoms of tRNA’s 3′-terminal adenosine (Figure 3H). The trapped AN2690-tRNA^Leu^ in the enzyme prevents further catalytic turnover, thus inhibiting the activity of LeuRS and consequentially blocking protein synthesis [74]. Therefore, in both cases, these editing site inhibitors still act their effect through inhibition of translation.

### 3.2. Dual Site Inhibitors

In order to efficiently transfer amino acids to tRNA, the three substrate-binding pockets of aaRSs (aa, ATP and tRNA-3′A76) are closely located. This topology provides an important strategy for developing higher affinity aaRS inhibitors by binding to more than one substrate site in aaRS. In fact, most high affinity aaRS inhibitors are found in this class (Table 1).

#### 3.2.1. ATP-Amino Acid Dual Site Inhibitors

**Figure 4:**
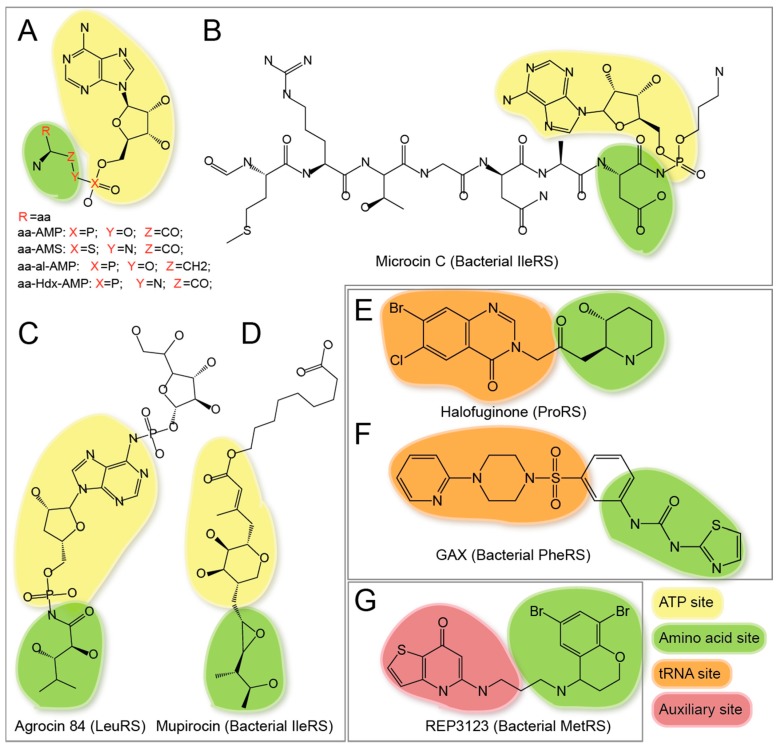
Dual site inhibitors of aaRS. (**A**–**D**) ATP-amino acid dual site inhibitors: (**A**) chemical structures of aminoacyl-adenylate (aa-AMP) and its analogues; (**B**) chemical structure of Microcin C; (**C**) chemical structure of Agrocin 84; and (**D**) chemical structure of mupirocin; (**E**–**F**) tRNA A76-amino acid dual site inhibitors: (**E**) chemical structures of ProRS inhibitor halofuginone (HF); (**F**) chemical structures of PheRS inhibitor GAX; and (**G**) chemical structure of the MetRS amino acid-auxiliary dual site inhibitor REP3123.

The amino acid sulfide adenylates (aa-AMS), aminoalkyl adenylates (aa-ol-AMP), and amino acid hydroxamate AMP (aa-Hdx-AMP) are non-hydrolyzable mimetics of aa-AMP, the reaction intermediate product of aaRS (Figure 4A) [128,129,130]. These compounds for each of the 20 aaRS families have been made. They probably form one largest (also the most complete) group of aaRS inhibitors [131]. These compounds can bind and inhibit the corresponding aaRS family but with little species specificity. They directly occupy and block the active site for ATP and amino acid with high affinity [131]. For example, the aa-AMS inhibitors display tight binding (Kd = ~ nM) to aaRS [132]. However due to the highly charged property of both amino acid and AMP moieties, aa-AMS inhibitors generally have poor penetration through the cell membrane [133].

Drug properties of aa-AMS may be improved by modifying the adenosine moiety. Using structure based drug design, a series of ThrRS inhibitors were successfully generated by replacing the adenosine of Thr-AMS with other fragments. Several compounds possess excellent binding affinities and greatly improved bacterial selectivity [87]. Modification of Leu-AMS into thiazole sulfametes also achieved over 300-fold selectivity for the *Escherichia coli* LeuRS *versus* the human LeuRS [88].

Modification of amino acid moiety into an appropriate short peptide can increase the cell permeability. Microcin C is a kind of Trojan-Horse antibiotic [89]. With an “MRTGNA” peptide linked to the N-terminus of the Asp residue (Figure 4B), Microcin C can be actively taken in bacterial cells including *Escherichia*, *Klebsiella*, *Salmonella*, *Shigella*, and *Proteus* species, and then processed by cellular aminopeptidases. The processed product is a non-hydrolyzable aspartyl-adenylate analogue that inhibits AspRS.

Agrocin 84 acts as a molecular Trojan-Horse by being processed inside the plant pathogen *Agrobacterium tumefaciens* into a toxic product TM84 (Figure 4C) [90]. Although as a Leu-AMP analog, TM84 cannot bind and inhibit LeuRS directly, but requires the tRNA^Leu^ to co-bind to the aminoacylation site [134]. TM84 traps the enzyme-tRNA complex in a novel “aminoacylation-like” conformation, with novel interactions between the KMSKS loop and the tRNA 3′-end. While targeting different site, TM84 and AN2690 are the only two aaRS inhibitors reported so far that are tRNA-dependent, and both inhibitors target LeuRS. Whether other aaRSs allow such tRNA-dependent inhibition remains unknown.

In addition to aa-AMS analogs and derivatives, mupirocin forms another type of ATP-amino acid dual site inhibitor [75]. It contains neither amino acid moiety nor adenosine moiety (Figure 4D), but structurally mimics Ile-AMP. The 3-methylbutan-2-ol moiety mimics Ile, the dihydroxytetrahydropyran ring mimics ribose, and the 3-methylbut-2-enoate part replaces the position of adenine. Mupirocin inhibits Gram-positive aerobic bacteria and a few Gram-negative strains through inhibition of IleRS. As the first approved aaRS inhibitor drug (approved in 1987), mupirocin’s selectivity for pathogenic IleRS over mammalian counterpart is about 8000-fold [135]. Interestingly, mupirocin binds to Ile-AMP binding site in the IleRS active site, which is very conserved through species. Only one residue difference (His581Asn) exists in the active site between the mupirocin-sensitive *Thermus thermophilus* IleRS and human enzyme. The His581 is 3.4 Å away from the mupirocin molecule. The Asn residue replacement may lose one hydrophobic interaction in human IleRS. Mupirocin analogues thiomarinol A and H were also found to be potent antibiotics [136,137]. Replacing the adenine moiety of Ile-AMS with the monate core structure of mupirocin generates the chimeric aminoacyl monate compound SB-234764, which has a Ki < 1 pM against *Staphylococcus aureus* IleRS and is the strongest reported aaRS inhibitor by far [138].

#### 3.2.2. Amino Acid-tRNA Dual Site Inhibitors

ProRS inhibitor HF is a low-toxicity, halogenated derivative of febrifugine (Figure 4E) [92]. Febrifugine is a bioactive natural product extracted from root of the hydrangea *Dichroa febrifuga* Lour used in traditional Chinese medicine [91]. HF is an amino acid-tRNA A76 dual site inhibitor. The piperidine ring structure inserts in the proline-binding pocket of ProRS. The halogenated 4-quinazolinone group is buried in the tRNA A76 binding site [76]. This dual-binding mode is also ATP-dependent. HF inhibits the ProRS translational activity of GluProRS in mammalian system *in vitro* [93], triggers amino acid response *in vivo* [68], inhibits T helper 17 (Th17) cells differentiation, and induces antifibrotic activities in fibroblasts [68]. Febrifugine has long been applied as an anti-parasitic agent, and the realization of its activity (as HF) as an anti-autoimmune response inhibitor suggests that aaRS inhibitors may be developed into therapeutic agents to treat human disease.

The phenyl-thiazolylurea-sulfonamides are a novel class of PheRS inhibitors. These compounds inhibit PheRS of gram-positive and gram-negative bacteria, with IC_50_’s in the nanomolar range and high specificity to the bacterial PheRS (α unit) than the mammalian cytoplasmic or the mitochondrial proteins [94]. One typical compound 1-(3-((4-pyridin-2-ylpiperazin-1-yl)sulfonyl)phenyl)-3-(1,3-thiazol-2-yl)urea (GAX) was recently co-crystallized with *Pseudomonas aeruginosa* PheRS (*Pa*PheRS) (Figure 4F). The structure shows the 1-phenyl-3-(thiazol-2-yl)urea moiety inserts into the Phe-binding site, while the 1-(pyridin-2-yl)piperazine moiety occupies the tRNA binding site [139]. The structure also suggested that the selectivity of the compound is possibly caused by two key interacting residues, Met99 (numbered by *Pa*PheRS) and Phe169. Both residues are conserved in bacterial PheRS, but Met99 is replaced by a Leu residue in mammalian cytoplasmic and mitochondrial homologues, and Phe169 is replaced by an Asn residue in mammalian cytoplasmic homologues.

Within the same amino acid-tRNA dual inhibitor group, HF and GAX both possess nanomolar inhibition constant against their targets but with different features. The interaction of HF with ProRS requires a specific conformation in the active center this is induced by ATP binding, while GAX inhibits PheRS in an ATP-independent mode as evidenced by the ATP-free PheRS/GAX complex structure.

#### 3.2.3. Amino Acid—“Auxiliary” Pocket Dual Site Inhibitors

REP3123 is a novel inhibitor of MetRS with ultra-high inhibition constant against *Clostridium difficile* MetRS (Ki: 20 pM) and >1000 fold selectivity over human mitochondrial and cytoplasmic MetRS [95]. Analogues of REP3132 were also found to be Gram-positive bacterial MetRS inhibitor [85,140,141]. Crystal structures show half of these compounds occupy the methionine binding pocket, and the other half bind to a so-called “auxiliary” pocket near by the methionine binding site (Figure 4G) [85]. These inhibitors can co-bind with ATP [141]. The binding of these compounds keeps MetRS in a different conformation comparing to the nature substrate binding state. Interestingly, binding of the auxiliary alone by a fragment of these compounds “2-aminoquinolin-8-ol” could trap MetRS in the new conformation [85]. Because *Clostridium difficile* is one major toxic bacteria in human intestine to cause infectious diarrhea, this inhibitor class serves as a potential therapy for *Clostridium difficile* infection [142].

There is no reported ATP-tRNA dual site aaRS inhibitor to our knowledge.

### 3.3. Triple Site Inhibitor

**Figure 5:**
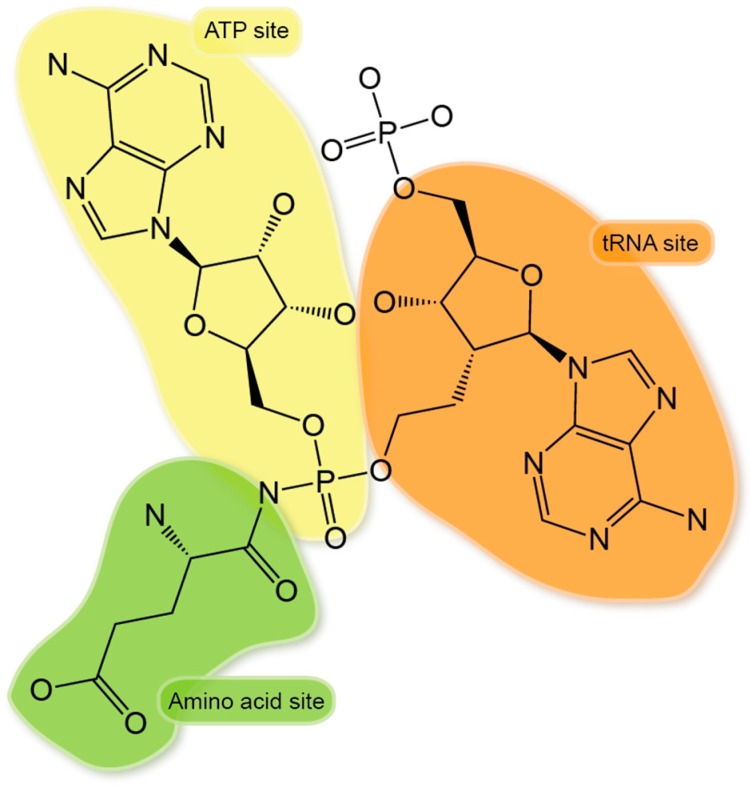
A conceptual triple active site inhibitor of aaRS. The structure shown here is a conceptual design of a triple active site inhibitor for GluRS. It contains a Glu-Hdx moiety (highlighted in green), an AMP moiety (highlighted in yellow), and a 2′ ethyl-linked AMP moiety, which mimics the A76 of tRNAGlu (highlighted in orange).

No triple active site (ATP-amino acid-tRNA) inhibitor has been reported. Several reasons may explain the rareness of triple active site inhibitors: (1) The dual active site inhibitor is potent enough. For example, the simple aa-AMS could easily obtain nM inhibition. (2) To occupy the three substrate-binding sites at the same time will significantly increase the complexity for chemical synthesis. However, the triple active site inhibitor may be more potent than other inhibitors, particular for certain aaRS families. For example, class I aaRSs ArgRS, GluRS, and GlnRS only catalyze the ATP/PPi pyrophosphate exchange reaction in the presence of the cognate tRNA, indicating a synergistic co-binding of the three substrates in these aaRSs [143,144]. The requirement for tRNA to co-bind may limit the potency of aa-AMS analogues to bind these three aaRSs. In fact, the affinity of Glu-AMS to GluRS decreased by 50-fold without tRNA^Glu^ [145]. A conceptual triple active site inhibitor with a tRNA-A76 like module plus an AMP and an amino acid-like moiety (Figure 5), as a full mimetic of the aminoacylation state, may give the most potent inhibition.

### 3.4. Multi Site Inhibitor

**Figure 6:**
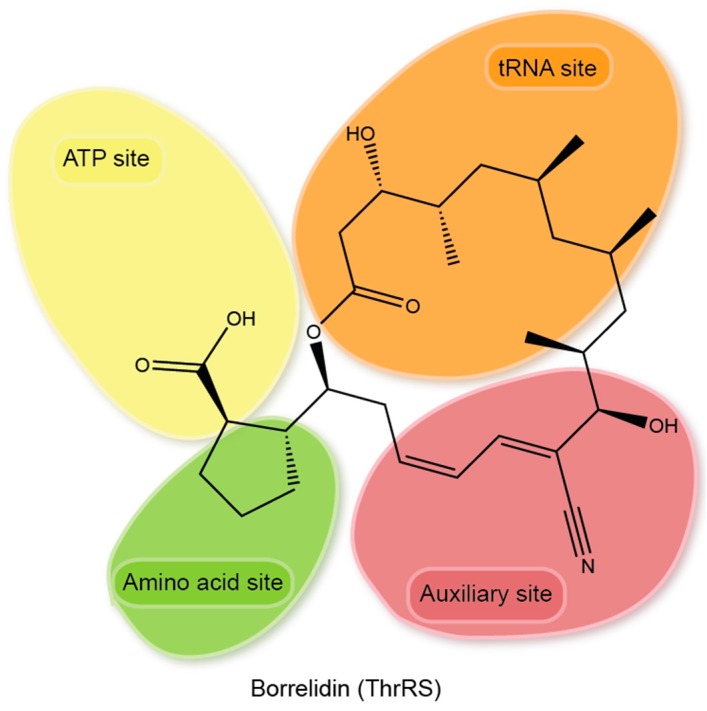
Multi-site inhibitor. Borrelidin uses a unique 18-membered ring structure binding to multi-sites at the active center of ThrRS.

Only one multi-site inhibitor (target more than three sites) was characterized so far. Borrelidin (BN) is a natural product isolated from *Streptomyces sp.* back in 1949 [146]. It blocks with sub-nM affinity to most bacterial and eukaryotic ThrRSs. Structural analysis reveals that BN excludes all three nature substrate from binding to the active center of ThrRS, in addition, occupies an extra space deep in the active center [96]. Unlike most other aaRS inhibitors, BN is an 18-member macrolide ring structure, sharing no structure similarity with ThrRS substrate. BN binds to ThrRS active center through an induced fit mechanism completely avoiding the substrate mimicking. BN is located at a joint key position in the active center of ThrRS, where the activation of amino acid happens (Figure 6). The cyclopentanecarboxylic group of BN clashes with the α-phosphate group of ATP and the carboxyl group of amino acid Thr. The additional polyketide C1–C3 region in the ring structure clashes with the A76 ribose of tRNA^Thr^. The rest of C4–C14 portion of the molecule wedges into a hydrophobic patch of the ThrRS, and induced the formation of an extra interaction pocket (the fourth site). Most of these BN-interacting residues are highly conserved in bacteria and eukaryotes, leading to the full-spectrum (bacteria and eukaryote) inhibition. In contrast, 12 of the 18 residues are altered in archaeal ThrRS, which is insensitive to BN inhibition.

Among 10 tested aaRS inhibitors including BN, Glu-AMS, Gln-AMS, Asn-AMS, Tyr-AMS, Ser-AMS, thialysine, mupirocin, cispentacin, and AN2729, BN showed the strongest inhibition on *P. falciparum* growth with an IC_50_ of 0.97 nM, which is more potent than the clinically used antimalarial artemether, artesunate, or chloroquine [97]. To reduce the cell toxicity to human cells, a library of BN derivatives was generated. Compound BC195 displays a 16,000-fold difference in inhibitory activity when tested on *P. falciparum* compared with human cells. And the most promising compound BC220 completely clears the parasitemia at 6 mg/kg per day, which is comparable to parasite clearance by chloroquine [97]. These results show BN supplies a promising scaffold for antimalarial drug design and validate ThrRS as a promising antimalarial drug target.

### 3.5. Non-Translational Function Inhibitor

Ongoing exploration of noncanonical functions of aaRS has shown important contributions to control of angiogenesis, inflammation, tumorigenesis, and other disease-related processes [27,38]. For example, the specific interaction between Gag and LysRS and its incorporation into the virion facilitate the HIV reverse transcription [147]. LysRS also induces cancer cell migration through interaction with the 67-kDa laminin receptor (67LR) [72]. ThrRS and TyrRS are reported to have pro-angiogenic functions [148,149]. Because angiogenesis is associated with cancer development, inhibition of the function of ThrRS and TyrRS in the control of angiogenesis may have some implications in tumor treatment.

**Figure 7:**
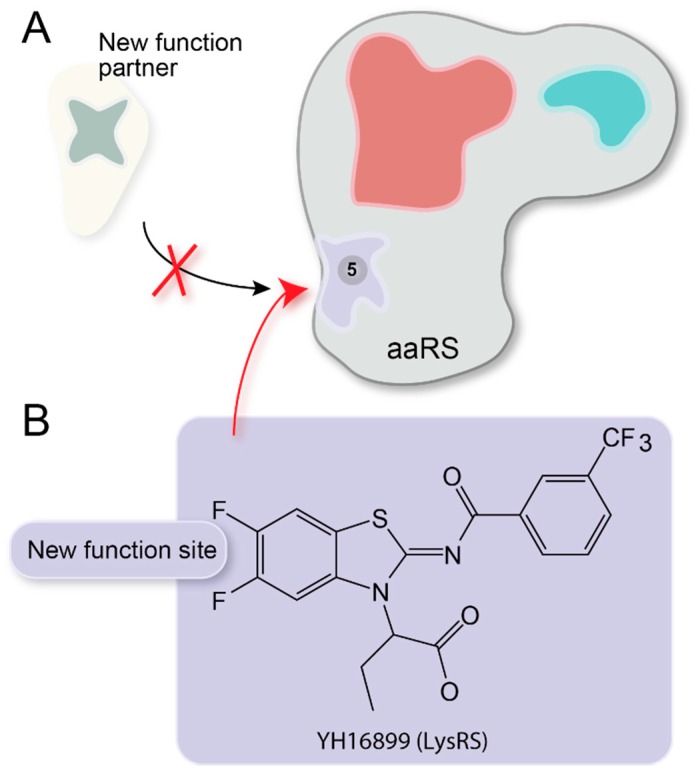
Non-translational function inhibitor. (**A**) Cartoon showing that inhibitors bind to the non-translational site of aaRS and block the interaction of aaRS to its new functional partner. (**B**) YH16899 is the first reported non-translational function inhibitor of aaRS. It binds to the anti-codon binding domain of human LysRS, blocks the interaction of LysRS to laminin receptor 67LR and inhibit the new function of LysRS in promoting cancer cell migration.

Small molecule has been validated to be able to bind aaRS and regulate the non-translational function completely independent of the translational active site center (Figure 7A). For example, the compound YH16899 binds to a site on human LysRS anticodon binding domain, blocks the interaction of LysRS to laminin receptor 67LR, thus inhibits laminin dependent cell migration of cancer cells (Figure 7B) [72]. In contrast to the numerous translational aaRS inhibitors, YH16899 is the only published non-translational aaRS inhibitor to date.

### 3.6. Other Ungrouped Inhibitors

A great number of developed aaRS inhibitors still do not have their mechanism of inhibition confirmed, waiting for being grouped into one of the five categories above (Table 1). For example, using structure-based *in silico* screening method, six and 11 compounds were selected as potential inhibitors against *P. falciparum* AlaRS (*Pf*AlaRS) and ThrRS (*Pf*ThrRS) aminoacylation site [98]. Among them, the *Pf*AlaRS inhibitor candidate 4-(2-nitro-l-propenyl)-1,2-benzenediol was able to inhibit *P. falciparum* 3D7 growth at a low IC_50_ value of 8 µM, while this compound did not show cytotoxicity against mouse L929 cells and human HeLa cells within the range of parasite IC_50_ values [98]. This compound provides a useful platform to generate second generation of antimalarial, though the mechanism of antimalarial activity of the chemical had not been confirmed to be through inhibition of AlaRS.

## 4. Perspective/Conclusions

Aminoacyl-tRNA synthetases, particularly human aaRS have been associated to various disorders (neurodegeneration, cancer, autoimmune diseases, *etc.*) with connections to both their translational function and functions beyond translation. Development of aaRS specific compounds will not only generate potential therapy for treating these human diseases, but also provide unprecedented tools to directly dissect the molecular mechanism of aaRS-related etiology and their unknown functions in biology.

All known aaRSs belong to total 24 families for the corresponding amino acid, and two classes for their evolutionarily distinct catalytic domain (Rossmann fold for class I and 7-strand beta-sheet for class II) (Figure 2A). Each of the two domains originated from a specific ATP-binding fold [150,151,152]. While different aaRSs catalyze the reaction of ATP with different amino acids, the organization of substrate binding sites in their active center is shared. The long-term evolutionary history of sequence divergence, combined with inherent constrain for protein synthesis, provides a powerful play station to allow inhibition of aaRS to be achieved. These intercalated conservation and diversities among aaRSs in different families and species, together as so-called “evolutionary limitations”, offer a broader ground for developing any desired modulator for aaRS. With more than six hundred structures of aaRSs and over one hundred inhibitors reported, these aaRS inhibitors can be classified by their binding modes into five categories: Single, Dual, Triple, Multiple site, and non-translational function inhibitors. In the future, new classes of aaRS inhibitors may appear, especially when parallel, far more efficient drug development campaigns for aaRS are launched.
